# Genetic and modifiable risk factors combine multiplicatively in common disease

**DOI:** 10.1007/s00392-022-02081-4

**Published:** 2022-08-20

**Authors:** Shichao Pang, Loic Yengo, Christopher P. Nelson, Felix Bourier, Lingyao Zeng, Ling Li, Thorsten Kessler, Jeanette Erdmann, Reedik Mägi, Kristi Läll, Andres Metspalu, Bertram Mueller-Myhsok, Nilesh J. Samani, Peter M. Visscher, Heribert Schunkert

**Affiliations:** 1 Department of Cardiology, Deutsches Herzzentrum München, Technische Universität München, Lazarettstr. 36, 80636 Munich, Germany; 2 Institute for Molecular Bioscience, University of Queensland, Brisbane, Australia; 3 Department of Cardiovascular Sciences, University of Leicester, Leicester, UK; 4 NIHR Leicester Biomedical Research Centre, Glenfield Hospital, Leicester, UK; 5 Deutsches Zentrum Ffür Herz- und Kreislauferkrankungen (DZHK), Partner Site Munich Heart Alliance, Munich, Germany; 6 Institute for Cardiogenetics, and University Heart Center, University of Lübeck, Lübeck, Germany; 7 DZHK (German Research Centre for Cardiovascular Research), Partner Site Hamburg/Lübeck/Kiel, Hamburg/Kiel/Lübeck, Germany; 8 Estonian Genome Centre, Institute of Genomics, University of Tartu, Tartu, Estonia; 9 Statistical Genetics, Max Planck Institute of Psychiatry, Munich, Germany; 10 Institute of Translational Medicine, University of Liverpool, Liverpool, UK; 11 Munich Cluster for Systems Neurology (SyNergy), Munich, Germany

**Keywords:** Coronary artery disease, Genome-wide association studies, Risk prediction, Risk score, Liability threshold

## Abstract

**Background:**

The joint contribution of genetic and environmental exposures to noncommunicable diseases is not well characterized.

**Objectives:**

We modeled the cumulative effects of common risk alleles and their prevalence variations with classical risk factors.

**Methods:**

We analyzed mathematically and statistically numbers and effect sizes of established risk alleles for coronary artery disease (CAD) and other conditions.

**Results:**

In UK Biobank, risk alleles counts in the lowest (175.4) and highest decile (205.7) of the distribution differed by only 16.9%, which nevertheless increased CAD prevalence 3.4-fold (*p* < 0.01). Irrespective of the affected gene, a single risk allele multiplied the effects of all others carried by a person, resulting in a 2.9-fold stronger effect size in the top versus the bottom decile (*p* < 0.01) and an exponential increase in risk (R > 0.94). Classical risk factors shifted effect sizes to the steep upslope of the logarithmic function linking risk allele numbers with CAD prevalence. Similar phenomena were observed in the Estonian Biobank and for risk alleles affecting diabetes mellitus, breast and prostate cancer.

**Conclusions:**

Alleles predisposing to common diseases can be carried safely in large numbers, but few additional ones lead to sharp risk increments. Here, we describe exponential functions by which risk alleles combine interchangeably but multiplicatively with each other and with modifiable risk factors to affect prevalence. Our data suggest that the biological systems underlying these diseases are modulated by hundreds of genes but become only fragile when a narrow window of total risk, irrespective of its genetic or environmental origins, has been passed.

**Graphical Abstract:**

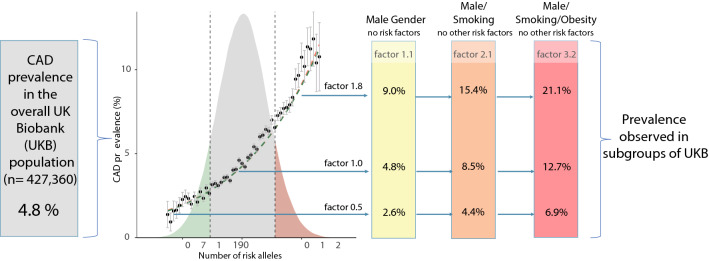

## Introduction

Most common noncommunicable diseases share a multifactorial etiology, with both exogenous and inherited factors contributing to their manifestation. In recent years, genome-wide association studies (GWAS) have substantially increased our understanding of the genetic component of disease risk. Specifically, hundreds of loci have been identified that all modulate risk of diseases such as coronary artery disease (CAD), breast cancer, prostate cancer, and type 2 diabetes mellitus (T2DM) as well as many other common diseases [1–4]. These findings have not only enriched the exploration of disease mechanisms but also raised the hope of improving risk prediction by assessment of the individual burden of risk alleles in form of genetic risk scores (GRS) [5–7]. However, from a clinical perspective, the conclusions from recent studies on this matter have appeared contradictory. Some found only small incremental benefits using GRS for risk prediction in the overall population whereas others identified individuals in whom a high GRS mirrored the risk of damaging mutations found in monogenic conditions [7–11].

Importantly, risk-conferring alleles are found with a high frequency in a given population. For example, three out of four Western-European ancestry individuals carry at least one risk allele at the 9p21 locus, which increases the odds of CAD by 1.23 [12]. Given the many genome-wide significant loci that have been identified in recent years [13], the total number of risk alleles carried by each person is likely to be very high. It is thus of critical importance to understand how these risk alleles interact. In the absence of empirical data, this interaction has been predicted by mathematical models, in which risk allele counts weighted by their estimated effect size from a GWAS are being combined. Examples are the logarithm of odds (Log) model or the Probit link function. Such widely used models predict the cumulative effects of risk alleles in a logistic function suggesting a sigmoidal relationship between GRS and probability of disease. Here, we conduct a systematic empirical evaluation of linear and multiplicative models to more precisely define risk conferred by common risk alleles [14–17].

From a clinical point of view, it is even more important—and not well defined yet—In other words, is the cumulative burden of common risk alleles tolerably low putting the population at the flat part of the curve—or beyond a turning point, where a few further multipliers (risk alleles) may cause a steep increase in disease prevalence?

Finally, factors such as smoking, T2DM, or obesity all increase prevalence of CAD and other common diseases. To better understand the principle mechanisms underlying the combinatorial effects of these and other risk factors with the genetic components, we simplified our analysis in that we focused on the bare number of highly significant risk alleles, albeit a genetic score built on millions of variants might be more precise in giving weight to the genetic risk conferred by each percentile of a GRS [7, 18].

## Methods

### Study participants

The UK Biobank project (UKB; http://www.ukbiobank.ac.uk) is a large prospective cohort study of ~ 500,000 individuals from across the United Kingdom, aged 40–69 years (56.4 ± 8.0) at the recruitment visit between 2006 and 2010 [19]. The flowchart of our analysis is shown in Supplementary Figure S1A. Following informed consent, health-related information was collected for each participant. In addition to self-reported information, dietary and exercise habits, multiple physical, cognitive and biochemical measurements were obtained. After quality control, we included 465,910 European individuals (Supplementary Figure S1B). CAD was defined as fatal or non-fatal myocardial infarction, percutaneous coronary intervention or coronary artery bypass grafting. ICD codes used for definition are listed in Supplementary Table S1. Breast cancer was defined by the primary and secondary ICD-10 diagnosis codes of “C50 Malignant neoplasm of the mammary gland”. Prostate cancer was defined by the primary and secondary ICD-10 diagnosis codes of “C61 Malignant neoplasm of the prostate”.

Since the diabetes GWAS, which led to identification of most of the genome-wide significant SNPs, included the summary statistics of UK biobank data, we used an independent source—Estonian biobank—for analysis of diabetes. The Estonian Biobank is a population-based cohort of the Estonian Genome Center at the Institute of Genomics of the University of Tartu [20]. Quality control details are found in the Supplementary Methods. We used 90,976 individuals for studying CAD and 91,195 individuals for studying T2DM. T2DM was defined by respective ICD-10 codes “E11 Type 2 Diabetes mellitus”.

None of the study participants was included in the GWAS that led to the identification of the risk alleles. All disease cases were considered for this study either being prevalent at recruitment in UKB or in the Estonian Biobank (fatal and non-fatal) or being registered during follow-up of the cohorts.

### Selection of risk variants

CAD, breast cancer, prostate cancer and T2DM were selected to represent common diseases each being affected by risk alleles at more than 100 autosomal loci discovered by GWAS meta-analyses [2, 3, 21]. GWAS summary statistics used for calculation are listed in Supplementary Tables S2–S5 and Figure S1C. Moreover, we calculated the genome-wide polygenic risk score using 6.6 million CAD SNPs [7].

### Genetic risk score and subgroups risk calculation

Individuals were grouped simply by the number of risk alleles to obtain their frequency distribution in the population in form of deciles, without giving weight to their respective effect sizes. The lowest decile was used as a reference. Missing genotypes were replaced by the expected value, which is twice the risk allele frequency. As a sensitivity analysis, we also constituted deciles of a weighted genetic risk score (wGRS), which was calculated based on risk variants by summing up the number of risk alleles weighted by the corresponding log odds ratio for the risk allele. All data on the wGRS are shown in the supplement. The analyses investigating the exchangeability of risk allele profiles are described in detail in the Supplementary Methods and Figure S2. The non-lipid CAD SNPs and lipid CAD SNPs were listed in Supplementary Table S6 [1].

### Stratification by modifiable risk factors

CAD prevalence was determined in each decile separated for exposure to smoking (ever versus never smokers), obesity (BMI ≥ 30 kg/m^2^), T2DM, or sedentary lifestyle (< 7.5 MET-h), detailed in the Supplementary Methods. Prediabetes was also classified as diabetes mellitus in this study, if participants indicated by questionnaire to have diabetes. Breast cancer prevalence was determined in each decile separated by obesity and alcohol intake for postmenopausal women. Finally, prostate cancer prevalence was quantified for men with and without a positive family history for prostate cancer and T2DM prevalence was quantified for people with and without obesity.

### Analysis of data and statistical methods

We assessed the goodness-of-fit of different statistical models of the relationships between risk allele frequency (x-axis) and prevalence/prevalence per allele (y-axis). We tested the prevalence contribution of risk alleles in extreme groups by estimating the correlation (R) of four competing models: Linear, Log, Probit, and Logistic. Respective plots display prevalence (on the left) and prevalence mediated per single risk allele (on the right) in groups with sample sizes larger than 200 subjects (e.g., Figure 2A, B). Scripts are found in the Supplementary Methods.

Mean values were calculated as arithmetic averages and represented as mean ± standard error. A *p* value of < 0.05 was considered statistically significant. Trends across deciles were tested by linear regression.

We used R [22] (version 4.0.3) and statistic packages of tidyverse, data.table, varhandle and ggplot2 for statistical calculations. All further details for the methods part could be found in Supplementary Methods.

## Results

### Risk of CAD in relation to risk allele distribution in UKB

**Fig. 1:**
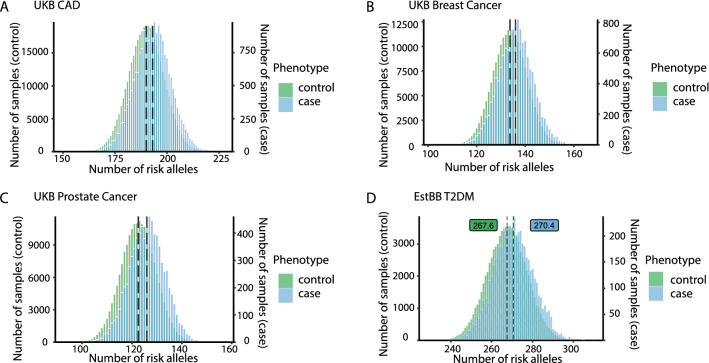
Histograms showing the distribution of risk alleles counts, which were normally distributed by Kolmogorov–Smirnov test (*p* values < 0.05), for coronary artery disease (CAD), breast cancer, prostate cancer, and type 2 diabetes mellitus (T2DM) in cases and controls separately. The number of common risk alleles per person were grouped in bins width of 2 risk alleles per person for respective diseases. Each person carried more than one hundred respective risk alleles with, on average, cases carrying 3–4 more than controls. Average numbers are shown for controls in green and for cases in blue boxes

In UKB, 427,360 subjects had phenotypic data regarding CAD, among whom we identified 20,310 cases. All risk alleles studied here were located on the diploid set of autosomal chromosomes such that the theoretical number of risk alleles at the 198 CAD risk loci we studied is between 0 and 396. Given the high frequency of the common risk alleles in the Western population we observed that a person carried an average of 190 CAD risk alleles. The mean number of CAD risk alleles per person was normally distributed (Fig. 1A) and varied between 175.4 and 205.2 in the bottom and top decile of the distribution, corresponding to a CAD prevalence of 2.4 and 8.2 percent, respectively (Table 1). When compared to the first decile, individuals in the tenth decile thus carried on average 29.8 or 16.9 percent more risk alleles whereas disease was 3.4-fold more prevalent (Table 1). An even wider spread in CAD prevalence was observed between the 1st (2.0 percent) and 99th (11.2 percent) percentile of the risk allele frequency distribution, which differed by 39.7 risk alleles (Supplementary Table S7). We used EstBB as an independent validation dataset and the results for CAD were similar to that in UKB (Supplementary Table S7, Table S8).

**Table 1:** Mean number of risk alleles and related disease prevalence in the 1st and 10th decile of risk allele distribution in the UKB traits (CAD, breast cancer and prostate cancer) and Estonian Biobank trait (T2DM)

Trait	Bottom decile of risk alleles in the population					Top decile of risk alleles in the population					Difference between the bottom and top decile		
	Alleles^#^ (*n*)	Cases (*n*)	Control (*n*)	Prevalence (%)	Prevalence per risk allele	Alleles^#^ (*n*)	Cases (*n*)	Control (*n*)	Prevalence (%)	Prevalence per risk allele (%)	Alleles (fold increase)	Disease risk (fold increase)	Prevalence per risk allele (fold increase)
CAD	175.4 ± 3.5	1042	41,691	2.44	0.014	205.2 ± 3.5	3487	39,244	8.16	0.040	1.17	3.3	2.9
Breast cancer	121.6 ± 2.9	720	21,159	3.29	0.027	146.1 ± 2.9	2208	19,668	10.09	0.069	1.20	3.1	2.6
Prostate cancer	110.4 ± 2.9	327	20,300	1.59	0.014	135.2 ± 2.9	1634	18,684	8.04	0.059	1.22	5.1	4.1
T2DM	250.9 ± 4.0	306	9124	3.24	0.013	285.1 ± 3.9	801	7784	9.33	0.033	1.13	2.9	2.5

^#^Average number of genome-wide significant risk alleles in cases and controls combined for the respective decile; Supplementary Table S8 displays the data across all deciles

### Generalizability to other common diseases

Studying 218,781 women and 205,624 men in UKB, we next determined all cases of validated breast (*n* = 13,221) and prostate cancer (*n* = 7832). For both diseases, the 134 published [2, 3] genome-wide significant risk variants were normally distributed (Fig. 1B, C). In the lowest decile of the frequency distribution, the prevalence of breast cancer and prostate cancer were 3.3 percent and 1.6 percent, respectively, with average numbers of risk alleles in these groups being 121.6 (breast cancer) and 110.4 (prostate cancer) (Table 1). Individuals in the top decile carried on average between 20.2 (breast cancer) and 22.4 percent (prostate cancer) more risk alleles whereas the disease prevalence went up by 3.1- and 5.1-fold, respectively (Table 1). In the lowest percentile of the risk allele frequency distribution, a low prevalence of breast cancer (2.3 percent) and prostate cancer (1.0 percent) contrasted with 14.0 and 11.2 percent in the 99^th^ top percentile (Supplementary Table S7).

We carried out the same analyses for T2DM in the Estonian Biobank (301 genome-wide significant risk variants, 91,195 individuals, Fig. 1D). In the lowest decile of the frequency distribution, the prevalence of T2DM was 3.2 percent with the average number of risk alleles per person being 250.9 (Table 1). Individuals in the top decile carried on average 34.2 more risk alleles whereas the disease prevalence went up to 9.3 percent (Table 1). In the 1st percentile of the risk allele frequency distribution, the prevalence of T2DM at 2.1 percent contrasted with 10.4 percent in the 99^th^ percentile (Supplementary Table S7). Respective data for the wGRS are shown in Supplementary Table S8 and Supplementary Figure S3.

### Per risk allele prevalence

**Fig. 2:**
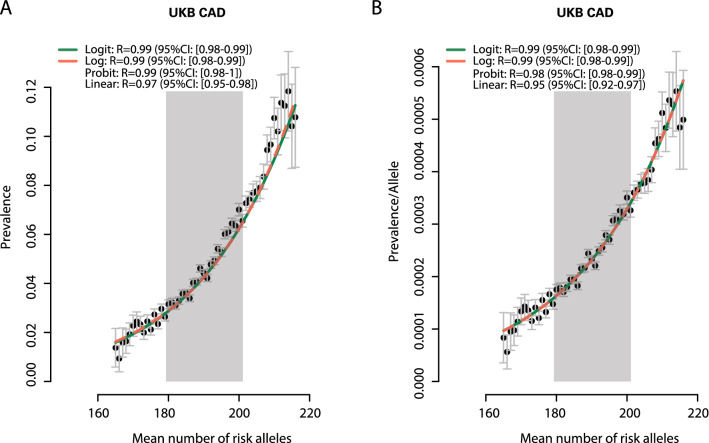
**A** Disease prevalence in relation to the number of risk alleles. The *Y*-axis displays the prevalence of coronary artery disease (CAD) in the UKB population. The *X*-axis displays the cumulative number of risk alleles per person. The correlation (R) between observed and predicted prevalence is given for each of four fitted functions, with its 95% confidence interval. **B** We divided the prevalence of each disease by the number of risk alleles per person, showing the effect of a single risk allele depending on a person’s overall burden of risk variants. The parts of the population residing between the 2nd and 9th decile of allele count distribution are highlighted in grey. The green and red lines show the fit from the logit and logarithmic functions, respectively

We next grouped subjects carrying the same number of risk alleles for a disease and determined the respective disease prevalence in these groups. Consistently across all diseases tested, we observed a sharp increase in prevalence with a relatively small increase in the number of risk alleles (Fig. 2A, Supplementary Figure S4). To investigate prevalence instigated per single risk allele we also display prevalence divided by the number of respective risk alleles carried in a person (Fig. 2B, Supplementary Figure S4). Remarkably, any given risk allele in the tenth decile of the risk allele distribution conveyed 2.9-, 2.6-, 4.2-, and 2.5-fold stronger effects on CAD, breast cancer, prostate cancer, and T2DM manifestation, respectively, than the same allele in the first decile (all *p* ≤ 0.01). since these were similar across deciles (Supplementary Table S8). In Fig. 2, the area shaded in grey represents the majority of subjects in the population, i.e., the second to ninth decile. Respective figures for breast cancer, prostate cancer, T2DM and the respective data on wGRS are shown in Supplementary Figures S5. Figures for independent validation on EstBB for CAD using both numbers of risk alleles and wGRS are shown in Supplementary Figure S4 and S5.

### Empirical evaluation of regression models

To better understand how risk alleles interact in mathematical terms, we modeled the relationship between disease prevalence and risk allele counts. Using generalized linear (GLM) regression models, which require specifying a link function, we compared the goodness-of-fit of using a Linear, Logistic (Logit), Probit, and Log link function. The goodness-of-fit of each of these four models was assessed using the correlation between the observed prevalence and the predicted prevalence from the corresponding model (Fig. 2 and Supplementary Figures S4). Consistently, we found that our data best fit non-linear link functions (i.e., Logit, Probit or Log), of which none consistently outperformed the others across different diseases. This finding, likewise, was replicated when we used 6.6 million SNPs [7] to calculate the CAD wGRS in UKB. Expectedly, the discrimination of risk is slightly better, but the principle functions remained to be highly similar to those seen with the 198 top-ranked SNPs. (Supplementary Figure S5K, Supplementary Table S8).

### Relevance of risk allele function

The prevalence of traditional risk factors and use of lipid-lowering medication was only marginally different across the deciles of the GRS (Supplementary Table S9a). Exclusion of variants previously shown to affect lipid levels (*n* = 28) or blood pressure (*n* = 14) had no influence on the exponential appearance of the regression curves for increasing numbers of risk allele and CAD prevalence (Supplementary Figure S6, A, B, E, F and Supplementary Table S9, Supplementary Figure S6, C, D, G, H). Likewise, in 10,000 random sets of 99 CAD-associated SNPs (out of the 198 CAD risk variants), we observed highly similar regression curves for CAD prevalence (Supplementary Figure S6, A, B, C, D) with Logit- and Log-based models showing regression coefficients (R) of 0.96 on average, with little variability (Supplementary Figure S6 E, F). These data suggest that the cumulative number of risk alleles rather than specific combinations of risk alleles is the major determinant of genetic risk.

**Fig. 3:**
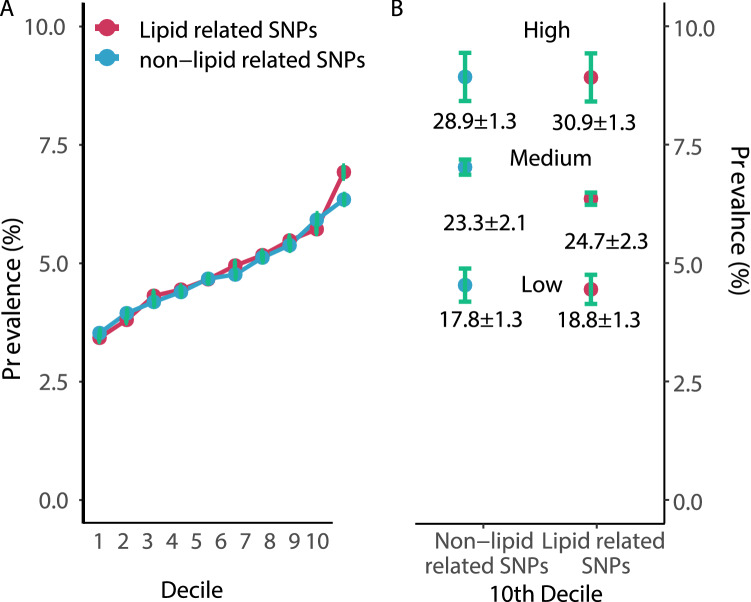
**A** Prevalence of coronary artery disease in relation to 28 risk alleles affecting lipid levels (red dots) and 28 risk alleles with equal odds ratios but no effects on lipids (blue dots). In the first decile individuals carried on average 18.9 ± 1.3 lipid-related risk alleles and 17.8 ± 1.3 non-lipid-related risk alleles. The respective numbers for the tenth decile were 30.9 ± 1.3 and 28.9 ± 1.3. **B **CAD prevalence is shown in subgroups of the tenth deciles of lipid and non-lipid SNPs. We subdivided subjects in the tenth decile of lipid-related SNPs in a high, medium, and low number non-lipid-related SNP subgroup. Vice versa, we subdivided the tenth decile of non-lipid-related SNPs according to high, medium and low numbers of lipid-associated variants. Top*, middle*, low* refers to the 10th decile, 2nd to 9th deciles and 1st decile. The effects of lipid-related and non-lipid-related risk alleles are interchangeable with respect to the prevalence of CAD

We next restricted the regression analysis to the 28 risk alleles known to affect lipid levels and compared their effects with 28 SNPs, which have similar published odds ratios for CAD but no effects on lipids (Supplementary Table S6). As shown in Fig. 3, the increase in CAD prevalence was similar across the ten deciles of the two groups of risk alleles. When we subdivided individuals within the tenth deciles of the two groups of risk alleles in those who carry low (first decile), medium (second to ninth deciles), or high (tenth decile) numbers of the risk alleles from the other respective group, the effects on disease prevalence remained comparable.

### Environmental risk factors and disease prevalence

**Fig. 4:**
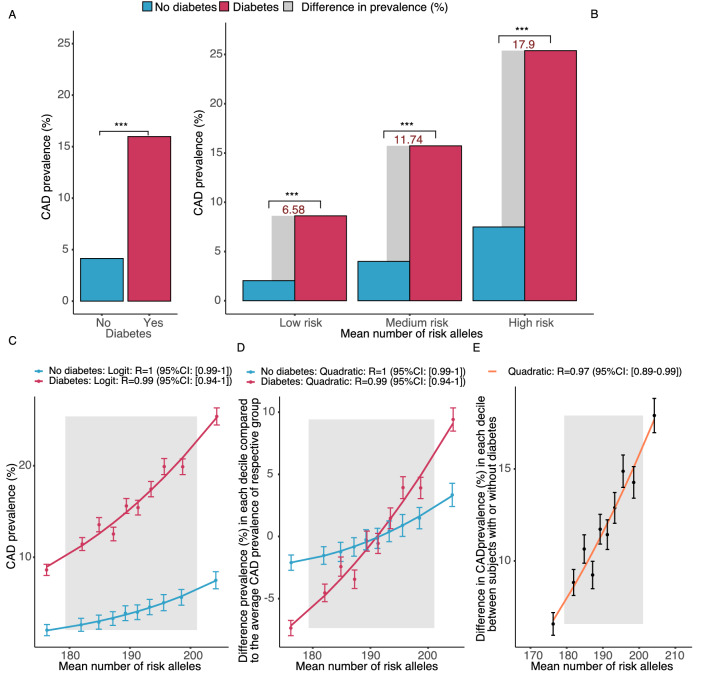
Prevalence of CAD in risk allele deciles with and without diabetes (other risk factors are shown in Supplementary Figure S7). **A** shows the prevalence for CAD in individuals with and without diabetes in UKB. **B** shows CAD prevalence in low (1st), medium (2nd–9th) and high (10th) deciles of risk allele distribution in the UKB without and with diabetes. The grey bars represent the difference in prevalence related to diabetes in the three genetic subgroups. As can be seen, the effect of diabetes is much larger in subjects with a high burden of risk alleles. **C** shows disease prevalence across the deciles of risk alleles in subjects with (red line) and without diabetes (blue line). The correlation (R) between observed and predicted prevalence is given for fitted logit functions with their 95% confidence interval. **D** shows disease prevalence across the deciles of risk alleles in subjects with (red line) and without diabetes (blue line) as a deviation from the average in the respective group. In the diabetes group the increase in risk with increasing numbers of risk alleles is by far steeper (linear regression coefficient: diabetes 0.0058, no diabetes 0.0019). **E** displays the difference in prevalence between subjects with diabetes and without diabetes across increasing deciles of risk alleles indicating that increasing risk alleles numbers enhance the effect of diabetes. In **D** and **E**, the correlation (R) between observed and predicted prevalence is given for a quadratic function, with its 95% confidence interval

We next studied the impact of established risk factors for CAD in UKB; Fig. 4A–D shows data on diabetes as an example. As compared to the first decile of the risk allele distribution, in the tenth decile, we observed that the absolute increases in prevalence related to diabetes, smoking, obesity, sedentary lifestyle, male sex, age ≥ 55 years, average total household income before tax below 18,000, being without cholesterol medication, being without blood pressure medication, and high cholesterol level (> 6.18 mmol/L) were 2.7, 3.0, 3.0, 3.8, 3.4, 2.9, 1.0, 5.4, 2.8, and 1.4-fold higher, respectively (all *p* < 0.01 for first vs the tenth decile; Fig. 4B and Supplementary Figure S7). In other words, a high genetic risk amplified the absolute prevalence conveyed by the respective risk factor.

Figure 4C shows a much more pronounced change in CAD risk for diabetes as compared to non-diabetic individuals across the deciles of the risk allele distribution, in relation to the average risk in the respective group. Importantly, in subjects exposed to a risk factor, we observed a by far steeper increase in absolute disease risk with increasing numbers of risk alleles than individuals without the respective risk factor (panel D and E in Fig. 4 and Supplementary Figure S7). Respective data for the wGRS—either with 198 significant SNPs or 6.6 million SNPs—are consistent and also shown in the supplement (Supplementary Figure S8 and Supplementary Table S10).

Similar data were observed for established risk factors for breast cancer (obesity, alcohol consumption), prostate cancer (positive family history), and T2DM (obesity). The absolute increases in prevalence were 7.6- (obesity-breast cancer), 8.1- (positive family history-prostate cancer) and 1.8-fold higher (obesity-T2DM) in the tenth as compared to the first decile of respective risk allele distribution and the effect of alcohol intake on breast cancer risk was only apparent in women with at least moderate genetic susceptibility (all *p* < 0.01).

## Discussion

By studying how established risk alleles affect the prevalence of common diseases, we report two important findings. First, we observed that respective risk alleles lead to a steep increase in risk for CAD, breast cancer, prostate cancer, or type 2 diabetes mellitus despite only small changes in their overall numbers. Second, all individual risk factors—genetic and environmental—act multiplicatively and interchangeably, and cause disease once a crucial turning point of cumulative risk is being passed. Essentially, irrespective of which biological pathways are being affected by disease loci or the combination of risk alleles with environmental exposures, the only quantity that matters is the total burden of these factors which put a person on a logarithmic curve of increasing risk.

Our findings have implications for the application of polygenic risk scores in predicting risk as well as for the understanding of biological mechanisms leading to the manifestation of the respective diseases. Regarding the first and clinically relevant topic: consistent with the genetic sampling theory, the largely random allocation of chromosomal segments during meiosis results in a normal distribution of common risk alleles. While the range of this distribution increases when more loci are detected to be associated with a trait, it is remarkable to observe overall relatively little variation in the high numbers of risk alleles across a population. This may explain why the predictive value of polygenic risk scores has been considered to be low [8–10]. However, looking at the first and tenth decile of the population, we observe an increase of disease prevalence by up to 5.1-fold. Thus, the predictive value of polygenic risk scores seems to be clinically relevant mostly for those at the outer ends of the distribution curve. In fact, given the apparent exponential increase in risk, those who carry high numbers of common risk alleles may have risks comparable to people carrying damaging mutations otherwise found in monogenic conditions [7].

Our observations are best explained by a simple multiplicative model, whereby the effect of each risk allele is proportional to the cumulative burden of all other risk alleles carried in a person. The resulting non-linear function has long been postulated to account for the risk of recurrence of common diseases mediated by multiple genetic risk factors in relatives [23–26]. Similarly, empirical data on multiple environmental risk factors are consistent with a non-linear relationship between the number of risk factors and prevalence [14]. Consistent with these observations, the standard model to analyze case–control GWAS data is a logistic regression, and genetic risk scores are constructed accordingly [14, 27]. The present data dissect and test these models to unprecedented levels and show a remarkably good fit for multiplicativity and exchangeability of risk factors. Consistent with theoretical studies [28, 29], we could not distinguish between different kinds of similar multiplicative models, i.e., Log, Logit, or Probit.

Regarding the biological mechanisms leading to respective diseases, it is relevant to note that the most common risk alleles have no effects on protein structure but rather small effects on gene regulation. Thus, the diseases studied here seem to be largely driven by alterations of transcriptional activity. In other words, the cumulative number of common risk alleles in the population is in the hundreds, however, at a certain point, a few additional ones appear to destabilize a system of co-regulatory activity in various tissues which ultimately affects risk. Whereas at the low end of the frequency distributions, many risk alleles were tolerated with a lifetime disease prevalence of 2 percent or less, most individuals carried way more than this tolerable number of risk alleles. In summa, these appeared to disturb gene regulation to a degree that ultimately resulted in the high prevalence of CAD, T2DM, breast and prostate cancer, and likely other common diseases, particularly when modifiable risk factors come into play. Our data, therefore, provide empirical evidence for the liability threshold model of disease, whereby the combined effect of multiple risk variants with small effects jointly may create a steep increase in risk once a critical number is being passed [29, 30].

We found no evidence that CAD risk alleles affecting traditional risk factors such as hypercholesterolemia or hypertension, behaved any different from the vast majority of CAD risk alleles, which currently have neither established disease mechanisms nor specific treatments [1]. While this observation implies that the etiology of the diseases is more complex than thought, it also calls for additional strategies to lower the genetic risk of atherosclerosis and its complications. Indeed, the effects of risk alleles that increase lipids could be partially neutralized by a lower number of risk alleles with non-lipid-related effects. Vice versa, a lower count of lipid-related risk alleles was equally effective in lowering the risk of those carrying high numbers of non-lipid-related risk alleles. This is in line—and may explain—previous studies, which observed that LDL-cholesterol lowering is more effective in lowering cardiovascular risk in people with a high as compared to those with a low genetic risk score [31–34]. In other words, pharmacological neutralization of risk alleles that increase LDL-cholesterol leads to a more pronounced decrease in risk if the person carries overall a high number of risk alleles because cumulatively they increase risk exponentially. Not only is that risk higher in such individuals, but their benefit from therapy also appears to be higher as well, explaining by far lower numbers needed to treat for preventing events in people with a high genetic risk score[31–34]. Indirectly, the data also add genetic evidence for guidelines on primary prevention that recommend lipid-lowering treatment in subjects having high overall risk despite LDL-cholesterol levels way below the population average [35, 36].

Risk alleles exerted multiplicative effects also with exogenous risk factors, i.e. the more risk alleles a person carried the stronger were the risks associated with smoking, obesity or lack of physical exercise. Vice versa, in people free of risk factors, we observed relatively little differences in disease prevalence across the distribution spectrum of risk alleles. As an example, the increase of absolute CAD prevalence related to the increase in genetic risk between the first and tenth decile of the risk allele distribution was 1.69% in non-smoking, non-obese women whereas it was 21.1% for male smokers with obesity.

Our study has several limitations. Most importantly, we aimed at elucidating the principles on how common risk alleles interact with each other and the prevalence variation with and without traditional risk factors rather than defining their precise weights. Further studies, based on the recent expansion of GWAS, meta-analyses may do this for each percentile of a GRS [37]. Indeed, GRS involving millions of SNPs will result in more precise estimates of genetic risk across the full spectrum found in the population (Supplementary Table S8) [7]. Our data indicate how such refined estimates for each percentile of a GRS can be used for adjustment of the absolute risk—based on individual age, gender, and risk factor profile—a person carries. Indeed, understanding the non-linear relationship on combined genetic and exposure risk factors will help to inform physicians regarding the groups of patients having the largest benefit from preventive treatments [38]. Next, both populations studied here as well as the previous identification of common risk alleles by GWAS have focused on individuals with Western-European descent, such that our findings may only apply to this ancestry group. However, the discovery of variants displaying genome-wide significant associations is still ongoing, such that more variants will be discovered, in particular in currently under-represented ancestry populations [39]. In this sense, our data aim to illustrate genetic principles rather than to offer definite risk estimates across populations. Furthermore, although we show that risk alleles act multiplicatively in diverse diseases, the generalizability to other common diseases and additional environmental risk factors needs to be verified. In addition, although we have shown a strong non-linear model fit between the risk allele burden and prevalence, we could not distinguish model fits between logarithmic, Logistic and Probit models, consistent with theoretical studies [40].

## Conclusion

In conclusion, we provide empirical evidence that genetic and non-genetic risk factors combine multiplicatively on prevalence and that they are exchangeable. Our results are consistent with gene–gene interactions and gene–environment relationship on the prevalence and a multiplicative model of liability to common disease, consistent with theoretical models that were proposed well before the GWAS era. Every person carries a large number of risk alleles yet a few more logarithmically increase disease prevalence explaining why the diseases we studied here are so common, a phenomenon that is largely exacerbated by modifiable risk factors. These findings offer a rationale for directing preventive efforts to individuals with a particularly high burden of combined genetic and non-genetic risk [31, 32, 41, 42].

## Abbreviations

SNP: Single-nucleotide polymorphism
CHR: Chromosome
BP: Base pair
OR: Odds Ratio
CAD: Coronary artery disease
T2DM: Type 2 diabetes mellitus
UKB: UK biobank
Est BB: Estonian biobank
wGRS: Weighted genetic risk score
